# Phenotypic and genotypic landscape of antibiotic resistance through One Health approach in Sri Lanka: A systematic review

**DOI:** 10.1111/tmi.14084

**Published:** 2025-01-06

**Authors:** Thilini Nisansala, Yasodhara Deepachandi Gunasekara, Nadisha Sewwandi Piyarathne

**Affiliations:** ^1^ Faculty of Veterinary Medicine Universiti Malaysia Kelantan Malaysia; ^2^ Asia‐Pacific Centre for Animal Health, Melbourne Veterinary School University of Melbourne Parkville Victoria Australia; ^3^ National Centre for Antimicrobial Stewardship, Melbourne Medical School University of Melbourne Victoria Australia; ^4^ Institute of Dentistry, School of Medicine Medical Sciences and Nutrition University of Aberdeen Aberdeen UK; ^5^ Department of Basic Sciences, Faculty of Dental Sciences University of Peradeniya Kandy Sri Lanka

**Keywords:** antibiotic resistance, One Health, Sri Lanka, systematic review

## Abstract

**Objectives:**

Antibiotic resistance (ABR) constitutes a significant burden to economies in developing countries. In the ‘One‐Health’ concept, ABR in human, animals, and environment is interconnected. The aim of this study was to critically appraise literature on ABR in all three domains in One Health, within the Sri Lankan geographical context.

**Methods:**

The protocol was registered with PROSPERO and followed PRISMA 2020 guidelines. A comprehensive electronic literature search was conducted in PubMed, Scopus, Web of Science databases and grey literature via Google Scholar. Out of 298 abstracts, 37 articles were selected following screening. A risk of bias assessment was conducted using Joanna Briggs Institute tools. Following blinded data extraction, descriptive data analysis and narrative synthesis were performed.

**Results:**

This review included studies published between 2016‐2023. Of the included studies, 17 (45.9%) reported data on samples obtained from humans, 9 (24.3%) from animals, and 6 (16.2%) from environmental sources, two studies (5.4%) from humans and animals, one study on animal and environment; whereas two studies including all three domains. ABR of 32 different bacteria (Gram negative⸺17, Gram positive⸺14) was retrieved; *E. coli* was the most frequently studied bacteria followed by MRSA and ESBL. For *E. coli*, a median resistance over 50% was reported for sulfamethoxazole (88.8%), trimethoprim (79.1%), ampicillin (60%) and tetracycline (50.3%) with the highest resistance for erythromycin (98%). Of a total of 21 antibiotic‐resistance genes in *E. coli*, the highest genotypic resistance was for tet‐A (48.5%).

**Conclusions:**

A comprehensive description of ABR for a total of 32 bacteria, 62 antibiotics and 46 ABR genes is presented. This review discusses the contemporary ABR landscape in Sri Lanka through the One Health lens, highlighting key methodological and empirical research gaps.

## INTRODUCTION

Antibiotic resistance (ABR) stands as one of the most pressing global health challenges of our time, significantly threatening human health, animal welfare, and the environment. The emergence and spread of ABR have the potential to undermine the effectiveness of antibiotics, leading to increased mortality rates, prolonged illnesses, and soaring healthcare costs. ABR and related conditions are responsible for 6.6 million deaths worldwide, making it the fourth leading cause of mortality [1].

A collaborative effort to mitigate this issue was spearheaded by the Food and Agriculture Organisation of the UN (FAO), World Health Organisation (WHO), the United Nations Environment Program (UNEP) and the World Organisation for Animal Health (WOAH, formerly known as OIE). Moreover, WHO announced a global action plan in the year 2015 to combat ABR [2]. Following up on this initiative, many nations implemented tailored action plans specific to their countries [3]. Addressing ABR risks at the intersection of human, animals, and the environment is an urgent priority. ‘One Health’ concept relies on a multidisciplinary approach, highlighting the need to systematically address this rapidly growing health challenge, to prevent the spread of ABR to nearby geographical regions via human travel and exchange of animal and environmental resources [4].

Sri Lanka is an island nation with approximately 20,000,000 population and is categorised as a lower‐ and middle‐income country by the World Bank [5]. In 2017, the Sri Lankan government published a 5‐year strategic plan to combat ABR [6]. In the post‐pandemic era following COVID‐19, inflation, high fuel prices and shortage of food supplies have led to economic instability; minimum importance has been applied to implement programs to combat ABR. This poses the risks of a rapid surge in ABR, cross‐domain spread, and transmission across geographies [7].

Regarding the antibiotic usage in Sri Lanka, a recent survey identified that total antibiotic consumption in the private sector was more than twice compared to the public health sector; macrolides, quinolones and other beta‐lactam antibacterials were the majority prescribed,while the Watch category antibacterials accounted for 46% of the total consumption [8]. Recent research findings in Sri Lanka have raised concerns on increasing ABR. A study by Perera et al. [9] reported ABR of Enterobacteriaceae causing both community‐acquired and hospital‐acquired urinary tract infections, where the highest prevalence was reported for Extended‐Spectrum Beta‐Lactamase (50%), followed by AmpC β‐lactamase (19%) and carbapenemase phenotypes (11%). Another study examining ABR in an ecosystem of organised livestock farming unveiled that among the tested *Escherichia coli* (*E. coli*), the highest resistance was observed against tetracycline, with a striking 51.7% resistance rate. Additionally, in the same study, resistance to ampicillin was reported at 39.4%, while nalidixic acid showed a resistance rate of 37.7% [10]. Similar ABR data was reported in bacteria found in water and soil samples collected from different ecosystems concerning the environmental domain [7, 11].

Gaining a comprehensive understanding of the phenotypic and genotypic aspects of contemporary ABR in Sri Lanka is crucial to develop evidence‐based strategies, identify research needs, review existing policy and guidelines to control and mitigate this issue effectively. There are no recent systematic reviews addressing the island‐wide ABR across human, animal and environmental domains. To address this research gap, the current study was conducted with the aim to systematically gather, summarise and synthesise evidence related to ABR data published after 2015, within the Sri Lankan geographical context.

## METHODOLOGY

### Protocol and registration

This systematic review was conducted in accordance with the PRISMA (Preferred Reporting Items for Systematic Reviews and Meta‐Analyses) version 2020 guidelines [12]. The research question was defined according to the SPIDER format (Sample: human, environmental and animal samples obtained from Sri Lanka, Phenomenon of Interest: ABR, Design: cross‐sectional, Evaluation: genotypic and phenotypic resistance, Research type: observational). The protocol was developed and registered with the PROSPERO: The International Prospective Register of Systematic Reviews with the registration number CRD42023388668.

### Data sources

A comprehensive systematic electronic literature search was conducted to identify relevant published studies using PubMed, Web of Science and Scopus databases. The reference lists of selected records were screened for further studies. A grey literature search was performed in Google Scholar that captured articles published in Sri Lanka Journals Online (SLJO) platform. The original literature search was conducted in December 2022, and the search was updated in July 2023.

### Search strategy

Keywords were combined with AND/OR Boolean to generate the search syntax. The search was conducted without time, language or any other restrictions by two reviewers independently (TN, YDG). Keywords used for the search were ‘Antimicrobial Resistance’ OR ‘Antibacterial resistance’ OR ‘Drug Resistance’ OR ‘Antimicrobial Susceptibility’ OR ‘Antibiotic Resistance’ OR ‘Antibiotic Susceptibility’ OR ‘Microbial Sensitivity Test’ OR ‘Drug Sensitivity Assay’ OR ‘ABR’ OR ‘Phenotypic Antimicrobial Resistance’ OR ‘Genotypic Antimicrobial Resistance’ AND ‘Sri Lanka’.

### Screening and study selection

Abstracts retrieved from the search were exported to EndNote library. Following de‐duplication, the records were uploaded to Rayyan.ai software for title and abstract screening. All records were screened by two blinded reviewers (TN and YDG). Following title and abstract screening, 85 records were selected for the full‐text screening stage by the same reviewers. Any disagreements during the selection process were resolved through discussion and involvement of a third reviewer (NSP). Study selection at all stages was conducted using the following predefined criteria.

#### Inclusion criteria

1. Original research (abstracts and full text) published in English
2. Study samples collected from Sri Lanka during and/or after 2015
3. Study samples including human, animal or animal‐originated food and environmental sources
4. Antimicrobial susceptibility test according to standard protocol for phenotypic interpretation and/ or genotypic studies conducted in a laboratory setting

#### Exclusion criteria

1. Publications including study design such as reviews, systematic reviews, meta‐analysis, case reports, case series and conference papers
2. Samples collected before 2015
3. Studies that have no laboratory data on genotypic or phenotypic analysis of ABR of samples
4. Data not presented for the individual bacterial species

**FIGURE 1:**
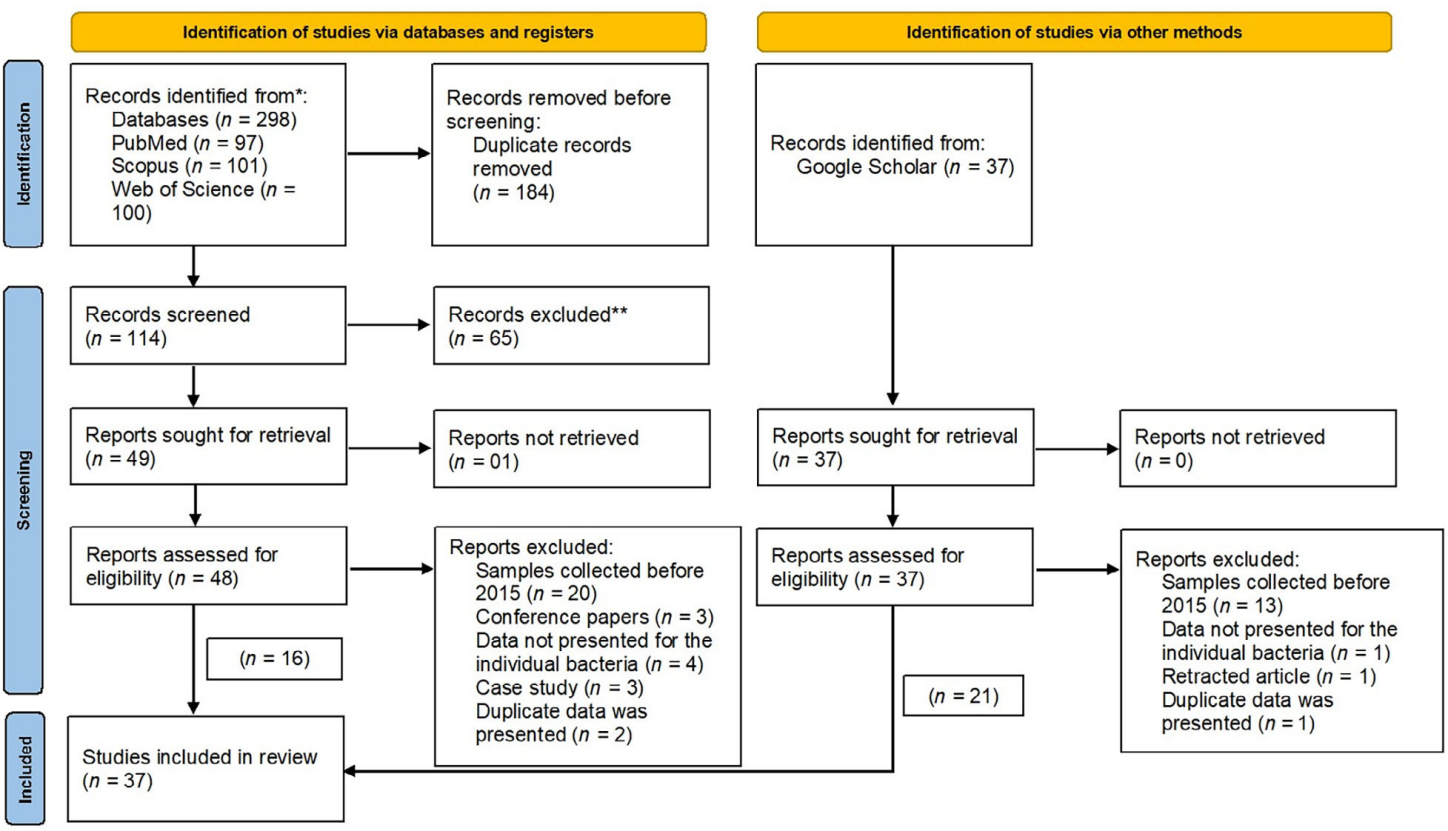
Study selection according to preferred reporting items for systematic reviews and meta‐analyses 2020 format.

Studies that presented data on samples collected before 2015 were excluded to restrict the results to contemporary research findings. Screening and study selection are summarised using PRISMA 2020 flow chart (Figure 1).

#### Reviewer calibration

Three reviewers (TN, YDG and NSP) extracted data, blinded to each other from five randomly selected papers for training and calibration. Once calibration was achieved, two reviewers extracted data from each paper independently and blinded to one another's scores. Two pairs of reviewers (NSP + TN, YDG + TN) conducted the data extraction. Disagreements were resolved through discussion and when necessary, with the involvement of a third reviewer (NSP or YDG).

### Data extraction

The variables extracted from the included articles were: first author, published year, year and duration of sample collection, sample category, study setting, type of sample, population of sample, clinical versus non‐clinical samples, sample size, species of bacteria, method of organism isolation and identification, laboratory methods, interpretation guidelines used for antimicrobial susceptibility testing, number and names of antibiotics tested and ABR genes analysed. The data were recorded and summarised using a customised Microsoft Excel spreadsheet.

### Data analysis

The sample types and research methodologies of the included studies exhibited significant diversity; hence a meta‐analysis was not considered. The results of phenotypic analysis were amalgamated. The outcomes were synthesised for bacterial organisms and antibiotics with more than three research studies with more than three data points using median percentages accompanied by the interquartile range (IQR), presented as figures. Box and Whisker plots were employed to represent data, wherein the boxes signify the IQR, and the whiskers denote the minimum and maximum data values. For other data, the results were tabulated as percentages. For genotypic analysis, the results were combined for bacteria and genes with more than three data points. The median prevalence was used as the summary measure. All statistical analyses and data visualisation were performed using Microsoft Excel 2013 and GraphPad Prism (version 8).

### Risk of bias assessment of evidence

Risk of bias assessment of all included studies was conducted using Joanna Briggs Institute (JBI) Critical Appraisal tools [13] by two independent reviewers (TN, YDG) blinded to each other's scores. Disagreements were resolved through discussion and via a third reviewer (NSP). A letter ‘Y’ was given to criteria that were satisfactory, ‘N’ was given to criteria that were not satisfactory and ‘U’ was given to criteria that were unclear. Studies with 7 or above ‘Y’ were graded with high quality. Studies with 6–5 ‘Y’ were graded with fair quality. Studies with 4 or less ‘Y’ were graded with poor quality.

## RESULTS

From the literature search, a total of 298 abstracts were retrieved. Following de‐duplication, 114 abstracts were subjected to title and abstract screening by application of exclusion criteria. Further, 37 articles were identified from grey literature search and other sources. Subsequently, 85 articles were subjected to full‐text screening. From these, 37 studies met the selection criteria and were included in the review.

### Characteristics of studies included in the data synthesis

Studies reported ABR data between 2016 and 2023 in Sri Lanka, with the majority (70.3%) during or before 2018. Of the included studies, 17 (45.9%) reported data on samples obtained from human, 9 (24.3%) from animals and 6 (16.2%) from environment sources. Two studies (5.4%) collected samples from both human and animals, one study from animal and environment, whereas two studies collected samples from all three domains. Sample size ranged from 9 to 900 with a median (IQR) of 134.5 (269). The characteristics of the included studies are described in Table 1.

**TABLE 1:** Characteristics of the studies included in the systematic review

Study reference	Sample category	Sample collection setting	Sample type	Sample size	Method/s of ABR analysis (phenotypic/genotypic)	Reported bacteria
Jayaweera and Kumbukgolla [14]	Human	Livestock farm	Nasal and axillary swabs from farmers	94	Phenotypic only	MSSA and MRSA
	Animal		Nasal or perineal or peri‐anal swabs from pig, cattle, poultry	188		
Karunanayake et al. [15]	Human	Hospital	NR	NR	Phenotypic only	*Neisseria meningitidis* and *Streptococcus pneumoniae*
Guruge et al. [16]	Environment	Hospitals, a lake and a wastewater canal	Water or/and wastewater	34	Phenotypic and genotypic	*E. coli*
Dhanapala et al. [17]	Animal	Aquaculture farm	Healthy fish: skin and mucous samples Diseased fish: kidneys, swabs from skin ulcers and/or fin rot (lesions)	Healthy fish–24 Disease fish–29	Phenotypic only	*Aeromonas* spp.
	Environment		Effluent water, pond sediment, sample of biofilm removed/scraped from aquarium tubing/pipes	24		
Zhu et al. [18]	Human	Hospital	Respiratory specimens (ET secretions and sputum) from ICU patients	379	Phenotypic and genotypic	Carbapenemase‐Producing *Klebsiella pneumoniae*
Tegner et al. [19]	Animal	Temples or archaeological sites frequented by local and international visitors	Fresh faecal pat from free‐ranging toque macaques (*Macaca sinica*) and tufted grey langurs (*Semnopithecus priam*)	98	Phenotypic only	*Campylobacter jejuni*, *Campylobacter coli* and *Salmonella enterica*
Fernando et al. [20]	Human	Hospital	Urine from UTI patients	61	Phenotypic only	ESBL (*E. coli* and *Klebsiella* spp.)
Liyanage and Manage [21]	Environment	Hospital environment	Effluent water and sediment samples from water ways in outside from hospitals	80	Phenotypic only	*Staphylococcus* spp., *Streptococcus* spp., *Micrococcus* spp., *Bacillus* spp., *Lactobacillus* spp., *Streptomyces* spp., *Acinetobacter* spp., *Enterobacter* spp., *E. coli*, *Moraxella* spp., *K. pneumoniae*, *Pseudomonas aeruginosa*, *Haemophilus influenzae*, *Aeromonas hydrophila*
Harshani et al. [22]	Animal	Milk collecting centres	Raw milk	NR	Phenotypic and genotypic	*Listeria monocytogenes*
Perera et al. [9]	Human	Hospital	Midstream urine samples from UTI patients	NR	Phenotypic and genotypic	*E. coli*, *K. pneumoniae* and Enterobacter
Ranasinghe et al. [23]	Animal	Retail shops and supermarkets	Chicken meat and edible chicken organs	250	Phenotypic only	*E. coli*
Priyadharshana et al. [24]	Human	Hospital and general practitioner's centres	Urine from UTI patients	465	Phenotypic and genotypic	ESBL (*E. coli* and *Klebsiella* spp.)
Keerthirathne et al. [25]	Human	Hospital	Broncho‐alveolar lavage from patients with pulmonary symptoms	150	Phenotypic only	Non‐tuberculous mycobacteria
Tissera et al. [26]	Human	Hospital	Respiratory tract specimens from ICU patients	56	Phenotypic and genotypic	ESBL (*E. coli* and *Klebsiella* spp.), *P. aeruginosa* and *Acinetobacter* spp.
Kumar et al. [27]	Environment	Community and hospital environment	Water from wastewater treatment plants and hospital influent, effluent, discharge point, canal water samples at hospital	9	Phenotypic only	*E. coli*
Sapugahawatte et al. [28]	Human	Hospital	Vaginal and peri‐rectal swabs from pregnant women Peri‐rectal swabs from neonates	539 (Pregnant women: pre‐delivery⸺250, post‐delivery 130, Neonates: 159)	Phenotypic and genotypic	*Streptococcus agalactiae*
Kulasooriya et al. [29]	Animal	Cultural event (The Festival of the Tooth)	Faecal samples from elephants	50	Phenotypic only	*E. coli* and *Salmonella* spp.
Senanayake et al. [30]	Human	Hospital	Pus/wound swabs, ear swabs, urine, sputum, tracheal aspirates, fluid aspirates and blood from patients	NR	Phenotypic only	MSSA and MRSA
Jayaweera et al. [31]	Animal	Dairy farms	Milk from mastitis cows	31	Phenotypic only	*E. coli*, *Klebsiella* spp. and *Staphylococcus* spp.
Medis et al. [32]	Human	Hospital	Peripheral blood, CVC blood or catheter tips at the time of routine removal from ICU patients	NR	Phenotypic only	Coagulase negative Staphylococcus
Pirashanna and Rajapaksha [33]	Animal	Veterinary hospital	Skin and ear swabs from dogs	30	Phenotypic only	*Staphylococcus* spp.
Weerakoon and Amarasekara [34]	Environment	Community	Water from Dug‐Well and piped tap line	10	Phenotypic and genotypic	*E. coli*
Kumar et al. [11]	Environment	River	Surface water samples	10	Phenotypic only	*E. coli*
Jayasundara and Amarasekara [35]	Environment	Community	Water from tap and well	10	Phenotypic and genotypic	*E. coli*
Bamunusinghage et al. [36]	Animal	National parks and sanctuaries	Faecal samples from wild, urban wild and livestock animals	165 (Wild⸺47, Urban wild⸺54, Livestock⸺64)	Phenotypic only	*E. coli*
Tay et al. [37]	Human	Hospital	Stool, blood, and joint fluid	NR	Genotypic only	Nontyphoidal *Salmonella enterica*
	Animal	NR	Raw chicken meat			
Priyantha et al. [38]	Animal	Big poultry integrators	Caecum of slaughtered broilers	NR	Phenotypic and genotypic	*E. coli*
Priyantha et al. [39]	Animal	Six regional laboratories and a large‐scale dairy farm	Milk from mastitis cows	NR	Phenotypic and genotypic	*E. coli* and Coagulase positive Staphylococcus
Meredith et al. [40]	Human	Hospital	Rectal swabs from post‐partum women and infants	398 (Mothers⸺199, Infants⸺199)	Genotypic only	*E. coli*
Munasinghe et al. [41]	Human	University	Nasal swabs and perianal swabs from university students	322	Phenotypic and genotypic	ESBL (*E. coli* and *Klebsiella* spp.), *S. aureus*, MSSA and MRSA
Kalupahana et al. [42]	Human	Pig farms and hospital	Nasal swab from farmers, farm employees and family members Human clinical MRSA isolates (Peradeniya Teaching hospital)	228	Phenotypic and genotypic	MRSA
	Animal		Nasal swabs from pigs	493		
	Environment		Dust samples from walls of pig pens	119		
Vidanapathirana et al. [43]	Human	Immunisation clinics and hospitals	Nasopharyngeal swabs from children attending immunisation clinics and being hospitalised with respiratory symptoms	900 (450 from each group)	Phenotypic only	*Streptococcus pneumoniae*
Nanayakkara et al. [44]	Human	Hospital	Low vaginal swab from pregnant women (pre and post‐delivery) Neonates⸺Peri‐rectal swabs	539 (Pregnant women: pre‐delivery⸺250, post‐delivery 130, Neonates: 159)	Phenotypic and genotypic	*E. coli* and *Klebsiella* spp.
Medis et al. [45]	Human	Hospital	CVC tips or blood from ICU Central venous catheter insitu	300	Genotypic only	Coagulase negative Staphylococcus
Dilrukshi et al. [46]	Human	Maternity clinics at hospital	Low vaginal and rectal swabs from pregnant women	175	Phenotypic and genotypic	*S. agalactiae*
Ariyawansa et al. [7]	Human	Hospital	Urine from UTI cultures	200	Phenotypic and genotypic	*E. coli*
	Animal	Broiler farms and aquaculture farms	Cloaca from broilers and shrimp	461 (Broilers⸺401, Shrimp⸺60)		
	Environment	Aquaculture farms	Pond water	93		
Chathuranga et al. [47]	Human	Hospital	Samples from patients with lower respiratory tract infections, skin and soft tissue infections or urinary tract infections from cancer patients	NR	Phenotypic only	*S. aureus*, *Pseudomonas* spp. and Enterobacteriaceae

Abbreviations: ABR, Antibiotic resistance; ESBL, Extended spectrum beta‐lactamases; ET, Endotracheal; ICU, Intensive care unit; MRSA, Methicillin‐resistant *Staphylococcus aureus*; MSSA, Methicillin‐sensitive *Staphylococcus aureus*; NR, Not reported; UTI, Urinary tract infection.

**FIGURE 2:**
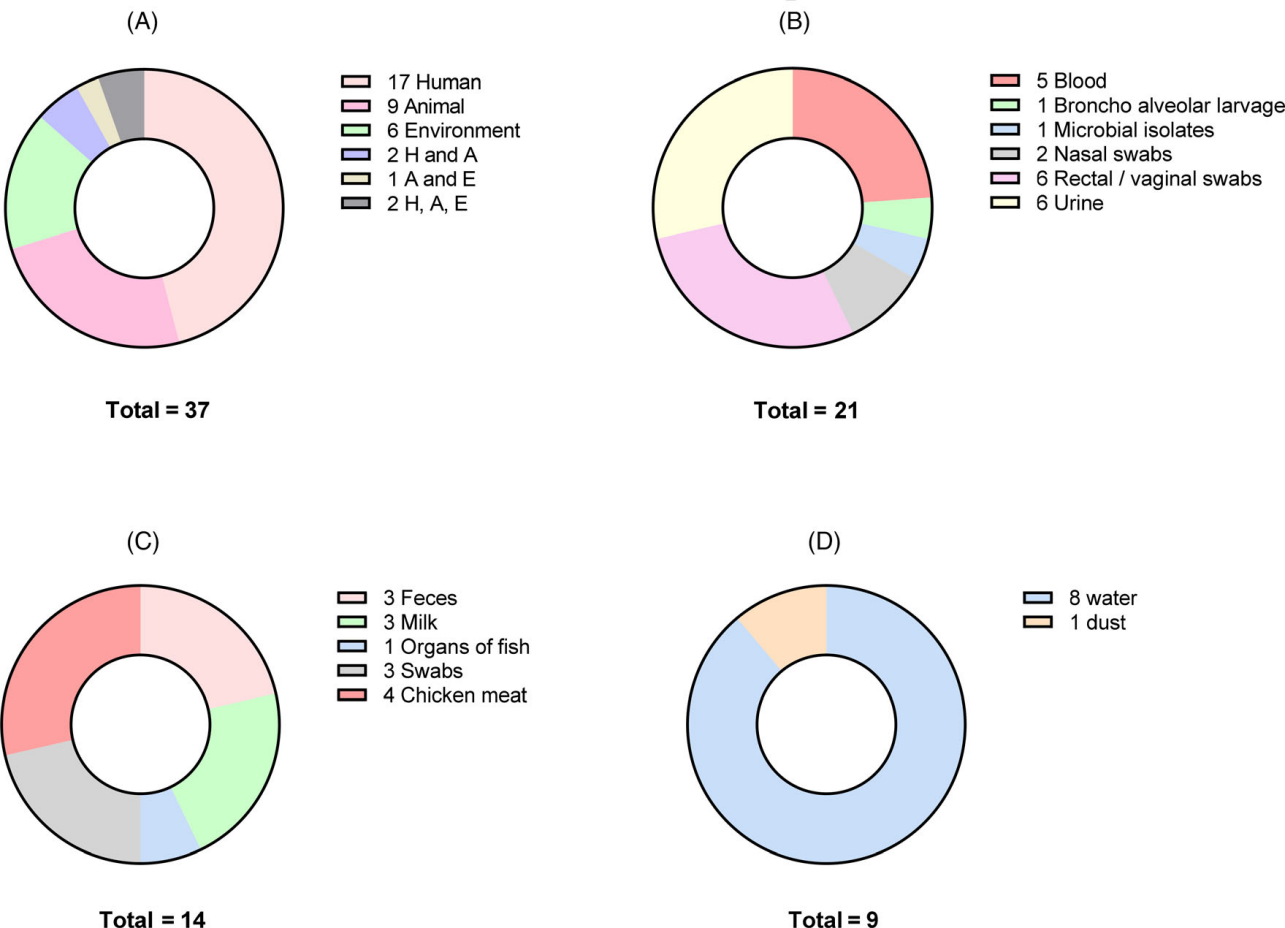
The distribution of studies and sample types across domains in the One Health concept (a) Distribution of studies according to domains, (b) Sample types in human studies, (c) Sample types in animal studies and (d) Sample types in environmental studies.

Out of the 21 studies reporting on human samples, 90.5% were from hospital settings. 62% (13) enrolled clinical samples and similar number of studies reported data for isolates from urine (6, 28.6%), rectal swab/perianal swab/stool (6, 28.6%), and blood (5, 23.8%). Four studies each (19%) obtained samples from ICU patients, pregnant women and patients with urinary tract infections, three (14.3%) studies collected samples from neonates and two (9.5%) studies collected samples from farmers. Among 14 studies reporting ABR on animal samples, 3 (21.4%) each collected faeces and milk, and the majority (11, 78.6%) were collected as non‐clinical samples. Five studies (35.7%) analyzed animal‐originated food, and 3 (21.5%) collected samples from farm animals. Other 6 studies collected samples from wild and companion animals. From the environmental studies, 8/9 collected effluent water from hospitals, farms and community, whereas 1 (11.1%) study utilised dust samples from walls of pig pens. The distribution of sample types across domains is presented in Figure 2.

### Methodological characteristics

Among all included studies, 19 (51.4%) reported data on phenotypic analysis, 3 (8.1%) genotypic analysis only, and 15 (40.5%) on both phenotypic and genotypic ABR. Among the phenotypic studies, 30 (88.2%) studies used the Clinical & Laboratory Standards Institute (CLSI) guidelines, and 3 (8.8%) studies used European Committee on Antimicrobial Susceptibility Testing (EUCAST) guidelines for result interpretation. For bacterial identification, more than half (23, 62.1%) of the studies used culture techniques only. Among the phenotypic studies, the majority (22, 64.7%) used disc diffusion method, while six (17.6%) studies used the minimum inhibitory concentration (MIC). In the genotypic studies, 14 (77.7%) used polymerase chain reaction (PCR) and 4 (22.2%) studies used whole genome sequencing (WGS).

### Phenotypic resistance

**FIGURE 3:**
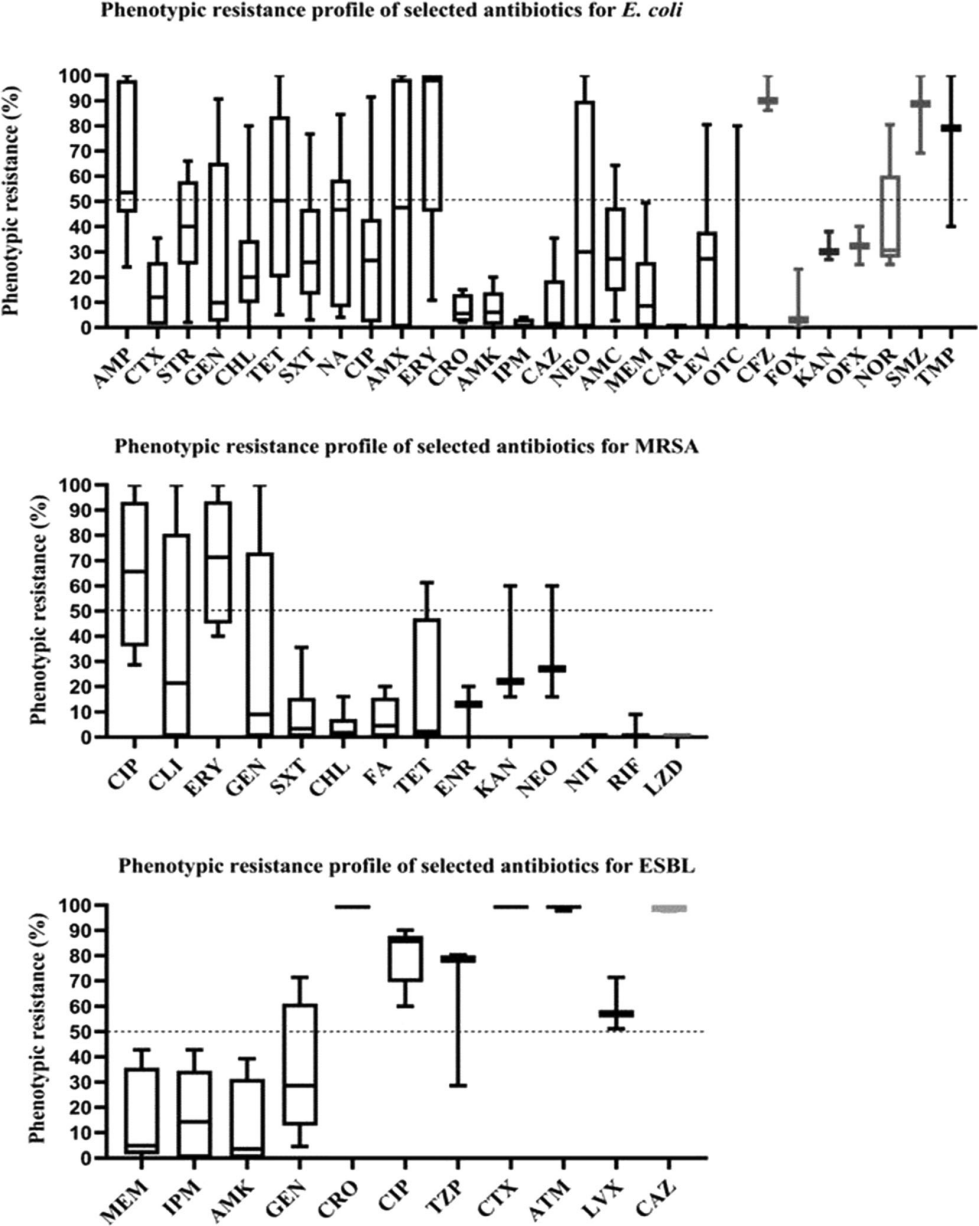
Phenotypic antibiotic resistance of *E. coli*, MRSA and ESBL to selected antibiotics. The boxplots in the figure represent the median and interquartile range (IQR) of phenotypic resistance reported for selected antibiotics. AMC, amoxicillin‐clavulanic acid; AMK, amikacin; AMP, ampicillin; AMX, amoxicillin; ATM, aztreonam; CAR, carbenicillin; CAZ, ceftazidime; CFZ, cefazolin; CHL, chloramphenicol; CIP, ciprofloxacin; CLI, clindamycin; CRO, ceftriaxone; CTX, cefotaxime; ENR, enrofloxacin; ERY, erythromycin; FA, fusidic acid; FOX, cefoxitin; GEN, gentamicin; IPM, imipenem; KAN, kanamycin; LEV, levofloxacin; LVX, levofloxacin; LZD, linezolid; MEM, meropenem; NA, nalidixic acid; NEO, neomycin; NIT, nitrofurantoin; NOR, norfloxacin; OFX, ofloxacin; OTC, oxytetracycline; RIF, rifampicin; SMZ, sulfamethoxazole; STR, streptomycin; SXT, trimethoprim‐sulfamethoxazole; TET, tetracycline; TMP, trimethoprim; TZP, piperacillin/tazobactam.

Among 34 studies that reported data on phenotypic analysis, there were 32 different bacteria (Gram negative⸺17, Gram positive⸺14). Data regarding the most frequently studied three bacteria, namely *E. coli*, MRSA and ESBL, are presented in Figure 3. The highest median phenotypic resistance rate for *E. coli* was reported for erythromycin (98%), followed by cefazolin (90%). *E. coli* exhibited a median resistance of over 50% for sulfamethoxazole (88.8%), trimethoprim (79.1%), ampicillin (60%) and tetracycline (50.3%). The lowest resistance for *E. coli* was observed against carbenicillin (0%) and imipenem (6%). For MRSA, the highest median resistance was observed for erythromycin (71.4%) followed by ciprofloxacin (65.6%), whereas three antibiotics showed low resistance of 0%, these were linezolid, nitrofurantoin and rifampicin. There were 7 antibiotics showing >50% of median resistance for ESBLs, namely ceftriaxone (100%), cefotaxime (100%), aztreonam (100%), ceftazidime (98.9%), ciprofloxacin (85.7%), piperacillin/tazobactam (78.6%) and levofloxacin (57.1%). Lowest resistance was observed towards amikacin (3.5%) and meropenem (4.9%) for ESBL (Figure 3).

The data not represented in Figure 3 are presented in Tables S1 and S2 (Supplimentary data files). Of Gram‐negative bacteria, only a few species of organisms exhibited complete phenotypic resistance to specific antibiotics. For *Acinetobacter* spp., the antibiotics cefotaxime, meropenem, ciprofloxacin, gentamycin, imipenem and piperacillin/tazobactam demonstrated 100% phenotypic resistance. Cefotaxime and ciprofloxacin showed 100% phenotypic resistance for Carbapenemase‐producing *Klebsiella pneumoniae* isolates. ESBL showed 100% phenotypic resistance towards ampicillin, *Haemophilus influenzae* and *Aeromonas hydrophila* towards tetracycline and *E. coli* towards cephalexin. Among Gram‐positive bacteria, 100% phenotypic resistance was demonstrated by MRSA towards cefoxitin and penicillin, MSSA towards clindamycin and *Streptococcus agalactiae* towards gentamycin. In a single study assessing phenotypic resistance among non‐tuberculous mycobacteria, it was determined that clarithromycin exhibited an 81% resistance rate, amikacin showed no resistance (0%) and ciprofloxacin had a resistance rate of 38.1%.

### Genotypic resistance

**FIGURE 4:**
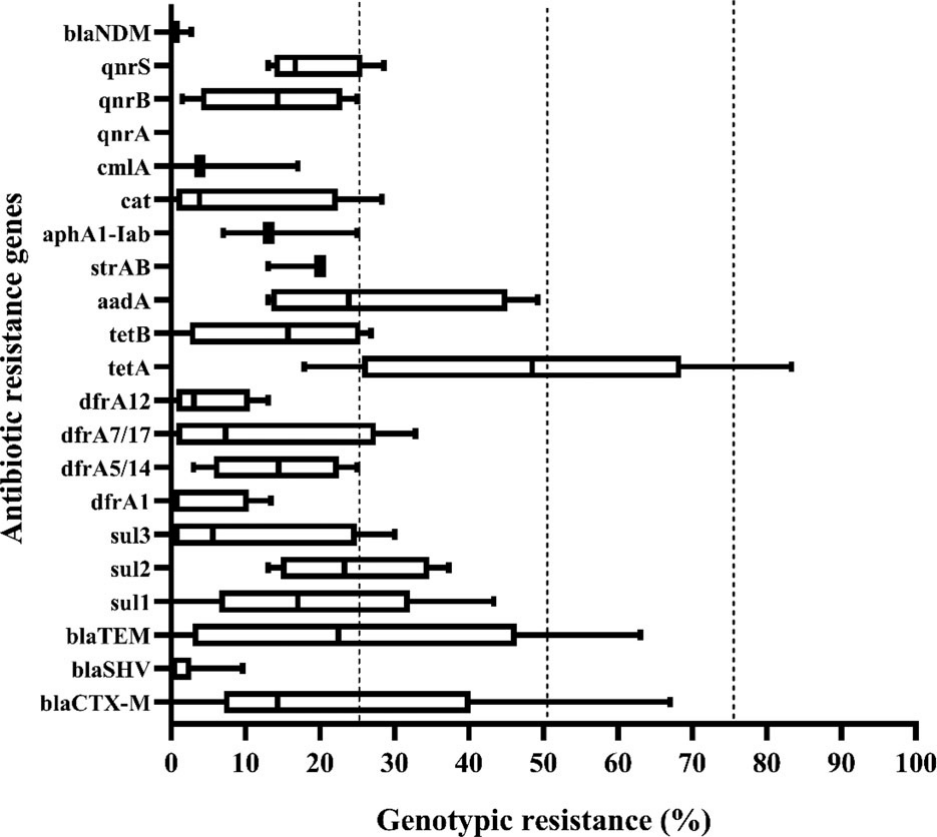
Genotypic antibiotic resistance of *E. coli* to selected antibiotic resistance genes. The boxplots in the figure represent the median and interquartile range of resistance reported if at least three data points were reported on the combination.

There were 12 different bacteria studied in the 18 genotypic studies (Gram negative⸺7, Gram positive⸺5). There was a total of 21 genes frequently reported for ABR of *E. coli*. All median percentages of genotypic resistance for *E. coli* were below 50%. The highest median resistance was observed for the tet‐A gene (48.5%), while the median genotypic resistance for all other genes was <25%. The lowest median genotypic resistance was found for the blaSHV gene (0%), followed by the blaNDM gene (0.38%). For ESBL, 100% genotypic resistance was observed for blaCTX‐M and blaSHV genes. Similarly, the blaCTX‐M, blaSHV, blaTEM and blaOXA genes reported 100% prevalence among carbapenemase‐producing *Klebsiella* spp. The genotypic resistance rates of the most frequently reported genes of *E. coli* are presented in Figure 4. The genotypic data not included in Figure 4 is presented Table S3.

### Risk of bias assessment

From the 37 included studies, 59.5% fulfilled more than half of the quality parameters used to assess the risk of bias of included articles (High: 5/37, 13.5%, Fair: 17/37, 45.9%), whereas 15 (40.5%) studies were categorised as poor with high risk of bias in results. Overall, the majority of studies included in this review were of fair (45.9%) and poor (40.5%) quality (Table 2).

**TABLE 2:** Risk of bias assessment of studies included in the review

Study reference	Was the sample frame appropriate to address the target population?	Were study participants sampled in an appropriate way?	Was the sample size adequate?	Were the study subjects and the setting described in detail?	Was the data analysis conducted with sufficient coverage of the identified sample?	Were valid methods used for the identification of the condition?	Was the condition measured in a standard, reliable way for all participants?	Was there appropriate statistical analysis?	Number of satisfied criteria (Y)	Quality grade
[14]	U	Y	U	Y	Y	Y	Y	Y	6	F
[15]	N	U	U	N	Y	Y	Y	Y	4	P
[16]	N	Y	U	Y	Y	Y	Y	Y	6	F
[17]	N	Y	N	Y	Y	Y	Y	Y	6	F
[18]	Y	N	U	Y	Y	Y	Y	Y	6	F
[19]	N	U	U	Y	Y	Y	Y	N	4	P
[20]	N	N	N	Y	Y	Y	Y	N	4	P
[21]	Y	U	U	Y	Y	Y	Y	Y	6	F
[22]	Y	N	U	N	Y	Y	Y	N	4	P
[9]	N	N	U	Y	Y	Y	Y	Y	5	F
[23]	Y	Y	Y	Y	Y	Y	Y	Y	8	H
[24]	Y	Y	Y	Y	Y	Y	Y	Y	8	H
[25]	U	U	U	Y	Y	Y	Y	N	4	P
[26]	U	N	N	N	Y	Y	Y	Y	4	P
[27]	N	Y	N	Y	Y	Y	Y	Y	6	F
[28]	Y	Y	N	Y	Y	Y	Y	Y	7	H
[29]	N	N	N	N	Y	Y	Y	N	3	P
[30]	N	N	N	N	Y	Y	Y	N	3	P
[31]	N	Y	N	Y	Y	Y	Y	Y	6	F
[32]	N	N	N	Y	Y	Y	Y	Y	5	F
[33]	N	N	N	N	Y	Y	Y	N	3	P
[34]	N	N	N	N	Y	Y	Y	N	3	P
[11]	N	Y	N	N	Y	Y	Y	Y	5	F
[35]	N	N	N	N	Y	Y	Y	N	3	P
[36]	N	Y	N	Y	Y	Y	Y	Y	6	F
[37]	N	N	N	N	Y	Y	Y	N	3	P
	N	Y	N	N	Y	Y	Y	N	4	P
[38]	N	N	N	N	Y	Y	Y	N	3	P
[39]	Y	Y	N	Y	Y	Y	Y	Y	7	H
[40]	N	Y	N	Y	Y	Y	Y	Y	6	F
[41]	Y	Y	N	Y	Y	Y	Y	N	6	F
[42]	N	Y	N	Y	Y	Y	Y	Y	6	F
[43]	Y	Y	N	Y	Y	Y	Y	Y	7	H
[44]	N	Y	N	N	Y	Y	Y	Y	5	F
[45]	N	Y	N	N	Y	Y	Y	Y	5	F
[46]	N	Y	N	Y	Y	Y	Y	N	5	F
[7]	N	N	N	N	Y	Y	Y	N	3	P
[47]	U	U	U	Y	Y	Y	Y	N	4	P

Abbreviations: Y, was given to criteria that were satisfactory. U, Unclear and N, was given to criteria that were not satisfactory, P, poor quality grade was assigned when the study satisfied 4 or less criteria; F, Fair quality grade was assigned when the study satisfied 6–5 criteria; H, High quality grade was assigned to studies that had 7 or more of satisfied criteria; NA, Not applicable.

## DISCUSSION

ABR is a significant cause of death around the globe, with an estimated 1.27 million deaths in the year 2019; in South Asia alone the death rate attributed to ABR was 21.5 (15.1–29.8) per 10,000 [1]. The ‘One Health’ concept denotes the significant interconnectedness between human, animals, and environmental domains: emphasising the need for holistic assessment and implementation of strategies to prevent the spread of ABR across domains. In developing nations, ABR can be aggravated significantly in the post‐pandemic era due to socioeconomic constrains. This is the first systematic review that critically appraises literature on ABR and its phenotypic and genotypic landscape, from data reported after the year 2015, in Sri Lanka under the One Health concept.

Despite being an escalating issue in developing nations, developed countries with well‐established healthcare infrastructures are also suffering from the ongoing ABR crisis. ABR poses an urgent and ongoing threat to the American healthcare system, carrying significant implications for public health. The growing resistance to life‐saving antibiotics necessitates a comprehensive examination of the factors driving this crisis and an assessment of the effectiveness of current strategies to address it [48]. Europe's ABR status under the One Health approach reflects significant progress but ongoing challenges. Through coordinated efforts across human, animals, and environmental health sectors, Europe has implemented robust surveillance, prevention and control measures to mitigate antimicrobial resistance. However, rising resistance of certain pathogens and the continued use of antibiotics in agriculture indicate that further action is needed to sustain progress and address cross‐border threats, effectively [49, 50, 51]. A recent systematic analysis with forecasts on the global burden shows that South Asia, Latin America and the Caribbean will have the highest all‐age mortality rates attibuted to antimicrobial resistance by 2050 [52].

Most of the research (*n* = 17, 45.9%) from the current review focused on human samples for ABR analysis. Similar observation was identified in reviews from Cameroon and Zambia, where majority of their studies enrolled biological samples from human subjects [53, 54]. In contrast, Vietnam reported majority (48.3%) as animal studies [55]. Due to the intricate nature of ABR, a holistic approach for simultaneous assessment across domains to contextualise it within the framework of the One Health approach is paramount, especially in low‐resource settings [56]. In this review, we identified only two studies (5.4%) reporting ABR on all three domains. Our results highlight the need for more research to be conducted with simultaneous assessment of ABR on humans, animals and environment to establish how ABR genes are circulated across domains. Further, a minimum number of studies assessed samples from the environment domain; this was identified as a major caveat indicating that environment is often overlooked as a prominent source of ABR transmission.

The characteristics of the included studies from Sri Lanka demonstrated a similar pattern compared to research conducted in African countries, Cameroon and Ethiopia. Majority collected samples from hospital settings [57, 58], most common samples collected from humans were urine, blood and swab samples [53] and among animal studies, majority enrolled farm animals or animal‐originated food [53, 58], while the most common environmental sample was effluent water from hospitals, farms and community [58]. The methods used to analyse ABR were similar to the methods reported in the current systematic review [53, 57, 58, 59].

ABR typically arises through genetic modifications, either by acquiring resistance genes or experiencing mutations. However, there are instances where resistance can emerge without any genetic changes, a phenomenon known as phenotypic resistance [60]. In our systematic review, most frequently studied organisms for phenotypic ABR were *E. coli*, MRSA, and ESBL. A recent systematic review with a focus on ABR of Enterobacteriaceae in Vietnam reported *E. coli* as the most common species analysed followed by *Salmonella* spp. [55], whereas a Zambian study reported *Staphylococcus aureus* as the most common organism being isolated followed by *E. coli* [54].

In the current review, more than 50% of phenotypic resistance rate for *E. coli* was reported for commonly used antibiotics such as erythromycin, cefazolin, sulfamethoxazole, trimethoprim, ampicillin and tetracycline. This result was in agreement with previously reported literature in majority of countries including Pakistan, Vietnam, India, Bangladesh, Nepal, Nigeria, Tanzania, Mozambique, Thailand, Portugal, Spain and China [61, 62]. According to data reported in Bangladesh, *E. coli* sourced from poultry environments were found to be phenotypically resistant to 14 different classes, including 45 different types of antibiotics [63]. The highest ABR was observed against penicillin followed by erythromycin, and ampicillin by a systematic review of *E. coli* isolated from water in Africa [64].

For MRSA, the highest median resistance was observed for erythromycin reported in Sri Lanka at 71.4% followed by ciprofloxacin at 65.6%. There is a substantial disease burden associated with MRSA resistance in Asia‐Pacific regions [65]. Many instances have been documented with its association with the highest global mortality rates [66]. A recent review indicated an elevated resistance to β‐lactam antimicrobials among MRSA isolates in Malaysia; during the 1990s and early 2000s, nearly all MRSA isolates in Malaysia exhibited resistance to erythromycin, with rates exceeding 90% together with low prevalence of clindamycin [67].

A total of seven antibiotics (ceftriaxone, cefotaxime, aztreonam, ceftazidime, ciprofloxacin, piperacillin/tazobactam and levofloxacin) demonstrated >50% of median resistance for ESBLs in Sri Lanka. Similar to our findings, a review in Nigeria reported that *E. coli* and *Klebsiella* spp. were the predominant ESBL organisms [68]. There were few other organisms namely *Acinetobacter* spp., Carbapenemase‐producing *K. pneumoniae, H. influenzae* and *A. hydrophila* that showed the highest resistance levels, and our findings were similar to other published data [69, 70, 71, 72].

It has been identified that several Gram‐positive bacteria, including MRSA, vancomycin‐resistant *Enterococcus faecium*, and drug‐resistant *Streptococcus pneumoniae*, pose significant threats by causing severe healthcare and community‐associated infections [73]. It is crucial to highlight that there is a limited number of studies conducted in Sri Lanka specifically addressing ABR in Gram‐positive bacteria, emphasising the pressing need for future research endeavours in this area.

The rise of ABR in bacteria is mostly due to their associated resistance genes. In our results, the genotypic landscape of ABR is presented for *E. coli* including 21 most frequently studied genes, where the most resistant of them was tet‐A. A review on the occurrence of ABR in *E. coli* in poultry and poultry environments in Bangladesh reported 24 different genes, and they observed a prevalence ranging from 1.2% to 100% [63]. Ramatla et al. [74] reported a consistently higher pooled prevalence of ABR genes (blaTEM‐M‐1, ampC, tet‐A and blaTEM) in *E. coli* in South Africa across animals, humans, and the environment domains.

For ESBL, 100% genotypic resistance was observed for blaCTX‐M and blaSHV genes and the blaCTX‐M, blaSHV, blaTEM and blaOXA genes reported 100% prevalence among carbapenemase‐producing *Klebsiella* spp. It was noted a similarly elevated incidence of resistance genes was consistent with findings in other studies including the countries from east, central, and Southern Africa, and Nepal [68, 75, 76, 77].

Our systematic review identified several common shortcomings of the research on ABR in Sri Lanka. Adequate size and representativeness of the sample to the population of interest is crucial when evaluating the ABR. There were significant lapses in sample size calculations and sampling techniques in the included studies, which contributed to a high risk of bias in results. Six leading bacterial pathogens were identified for deaths associated with ABR, naming *E. coli*, followed by *S. aureus*, *K. pneumoniae*, *S. pneumoniae*, *Acinetobacter baumannii* and *Pseudomonas aeruginosa* [1]. However, the results of the current systematic review revealed a remarkable need for further research to evaluate the ABR in *K. pneumoniae*, *S. pneumoniae*, *A. baumannii* and *P. aeruginosa* species as they were largely underexamined in the Sri Lankan context

Many studies did not specify whether the samples analysed were obtained from colonised individuals (non‐clinical) or animals or those with active infections (clinical samples). This distinction is critical, as colonisation often represents a reservoir of ABR without clinical disease, while infection requires treatment and is directly associated with clinical outcomes. The lack of differentiation may lead to an overestimation of the burden of ABR and misinterpretation of the data. These limitations can affect the development and implementation of targeted antimicrobial stewardship interventions.

Drinking water sources such as pipelines of community water supply, domestic wells in different geographical regions can provide critical data on how ABR is transmitted from environmental sources to humans. Our results revealed that effluent water and surface water from rivers contain significant bacterial ABR. It is recommended that rules and regulations should be strictly implemented to purify wastewater from hospitals and animal farms before releasing it into the common water sources such as rivers. Exploring ABR within the environmental sources surrounding feed and meat processing plants, slaughterhouses, and pharmaceutical companies is underexplored. These environments can serve as significant contributors to the dissemination and amplification of ABR due to the extensive use of antimicrobials in various industrial processes. Antibiotics are delivered to farms by wholesale, retail and via prescriptions. In Sri Lanka, most commonly used antibiotic in poultry farms was reported as Tylosine [7]. There was a scarcity of data for ABR of certain animal‐originated food sources such as different fish meat, pork, beef, lamb and eggs. Sri Lanka is recognised as a biological hotspot, boasting diverse ecosystems and a rich variety of wildlife. Hence, understanding the potential for ABR among wild animals is crucial, as they might serve as reservoirs and carriers of ABR. However, there is a notable scarcity of comprehensive research on this subject.

Although the overprescription of antibiotics by healthcare professionals and unregulated use in animal husbandry are significant contributors to the rise of ABR, the availability of reliable research data on these aspects in the Sri Lankan context is highly limited. It is noteworthy that, data on the proportion of antibiotics prescribed by medical, dental, and veterinary practitioners is scarce. Additionally, antibiotic usage in animals is not separately surveyed by species, and data on usage in the plant sector is negligible. Future studies should aim to collect and analyse such data for the better understanding of the contributions of different domains within the One Health framework in promoting ABR in Sri Lanka. A recent study reports that in human samples, resistance was highest to beta‐lactam antibiotics while in animal samples, resistance was highest to erythromycin [7]. A study investigating the reasons behind overprescription of antibiotics by clinicians reported that, in the Sri Lankan context, major contributors for antibiotic misuse were diagnostic uncertainty, and structural factors such as patient demand, hospital environment, patient poverty, limited testing facilities and the limited availability of antibiotics in hospitals [78]. Evidence based results from the current study indicate that most effective antibiotics for *E. coli* were carbenicillin and imipenem; for MRSA linezolid, nitrofurantoin, and rifampicin; and for ESBL linezolid, nitrofurantoin, and rifampicin.

The findings of this systematic review should be interpreted in light of its limitations. We excluded publications other than journal articles from the review, considering the validity of the findings; this may be a potential limitation concerning data reported in conference proceedings and unpublished data in theses. Another limitation of this study is the exclusion of theses and dissertations from the analysis. While the decision to focus on published literature was guided by considerations of accessibility and perceived quality, it is important to acknowledge that theses are rigorously reviewed academic documents. Excluding these sources may result in the omission of valuable insights and data, particularly from underrepresented regions or disciplines. Future reviews should consider incorporating such grey literature to provide a more comprehensive understanding of the topic.

We recommended implementing antibacterial stewardship programs across both medical and veterinary sectors to mitigate the escalating issue of ABR in Sri Lanka. In veterinary settings, such stewardship programs would regulate the judicious use of antibiotics in animal healthcare, agriculture and aquaculture. Similarly, in medical sectors, such programs advocate for optimal antibiotic prescribing practices among healthcare professionals, guided by contemporary research findings, emphasising the importance of accurate diagnosis, appropriate prescription and patient education to prevent misuse or overuse of antibiotics.

## CONCLUSIONS

ABR is a significant health issue with emerging disquiet in Sri Lanka. Transmission across human, environment and animal domains is a growing concern that needs immediate attention, and simultaneous assessment of ABR across domains is a necessity to comprehend ABR transmission. Our results revealed that a significant number of commonly prescribed antibiotics namely sulfamethoxazole, trimethoprim, ampicillin and tetracycline were more than 50% resistant to *E. coli*. The landscape of phenotypic ABR of a total number of 32 bacteria for 62 antibiotics, together with genotypic resistance of 46 genes, including 21 different genes of *E. coli* is compiled in this review. This review offers an in‐depth analysis of the contemporary ABR in Sri Lanka within the framework of the One Health concept, emphasising methodological and empirical gaps in existing research.

## FUNDING INFORMATION

This research was self‐funded. For the purpose of open access, the corresponding author's affiliated institute (University of Aberdeen) has applied a Creative Commons Attribution (CC BY) licence to any Author Accepted Manuscript version arising from this submission.

## CONFLICT OF INTEREST STATEMENT

Authors have no conflicts of interest to declare.

## Supporting information


**Table S1.** Phenotypic antibiotic resistance (%) of Gram‐negative bacteria to tested antibiotics.
**Table S2.** Phenotypic antibiotic resistance (%) of Gram‐positive bacteria to tested antibiotics.

**Table S3.** Genotypic antibiotic resistance.
