# Supplementary material for: Phenotypic and genotypic landscape of antibiotic resistance through One Health approach in Sri Lanka: A systematic review
Source: Trop Med Int Health. 2025 Jan 6;30(3):143–58. doi: 10.1111/tmi.14084 (PMC11873755; doi:10.1111/tmi.14084)
Supplement: Supplementary file 1 — Table S1. Phenotypic antibiotic resistance (%) of Gram‐negative bacteria to tested antibiotics. Table S2. Phenotypic antibiotic resistance (%) of Gram‐positive bacteria to tested antibiotics. [file TMI-30-143-s001.docx]

**Supplementary Data**

Supplementary Data Table S1: Phenotypic antibiotic resistance (%) of Gram-negative bacteria to tested antibiotics

| **Antibiotic** | ***Neisseria meningitidis*** | ***Aeromonas* spp.** | **Carbapenemase Producing**  ***Klebsiella* pneumoniae** | | ***Campylobacter* (*jejuni, coli*)** | ***Salmonella*** | **ESBL**  **(*E. coli* &/or *Klebsiella* spp.)** | ***E. coli*** | ***Acinetobacter* spp.** | ***Pseudomonas aeruginosa*** | ***Pseudomonas* spp.** | ***Enterobacter* spp.** | ***Moraxella* spp.** | ***Klebsiella pneumonia*** | ***Haemophilus influenza*** | ***Aeromonas hydrophila*** | ***Klebsiella* spp.** | **Enterobacteriaceae** |
| --- | --- | --- | --- | --- | --- | --- | --- | --- | --- | --- | --- | --- | --- | --- | --- | --- | --- | --- |
| Penicillin | 75 | NR | | NR | NR | NR | NR | NR | NR | NR | NR | NR | NR | NR | NR | NR | NR | NR |
| Levofloxacin | 37.5 | NR | | NR | NR | NR | Fig 3 | Fig 3 | NR | NR | NR | NR | NR | NR | NR | NR | 0  6.1  0 | NR |
| Cefotaxime | 0 | NR | | 100 | NR | 0  0 | Fig 3 | Fig 3 | 100 | NR | NR | NR | NR | NR | NR | NR | NR | NR |
| Meropenem | 0 | NR | | 70 | NR | NR | Fig 3 | Fig 3 | 100 | 13.3 | 12.9 | NR | NR | NR | NR | NR | 0  0 | 9.6 |
| Ciprofloxacin | 37.5 | NR | | 100 | 58 | 0 | Fig 3 | Fig 3 | 100  7.32 | 0 | 25.8 | 0 | 0 | 0 | 0 | 0 | 72.7  0  6.1  0 | 48.9 |
| Amoxycillin | NR | 97.5  94.3  89.5 | | NR | NR | NR | NR | Fig 3 | 36.59 | 35.29 | NR | 27.54 | 28.57 | 42.86 | 0 | 0 | NR | NR |
| Nalidixic acid | NR | 72.5  60  62.8 | | NR | NR | 0 | 88 | Fig 3 | NR | NR | NR | NR | NR | NR | NR | NR | NR | NR |
| Rifampicin | NR | 2.5  5.7  8.1 | | NR | NR | NR | NR | NR | NR | NR | NR | NR | NR | NR | NR | NR | NR | NR |
| Chloramphenicol | NR | 5  11.4  4.7 | | NR | NR | NR | NR | Fig 3 | NR | NR | NR | NR | NR | NR | NR | NR | 27.3 | NR |
| Doxycycline | NR | 12.5  31.4  17.4 | | NR | NR | NR | NR | 60 | NR | NR | NR | NR | NR | NR | NR | NR | NR | NR |
| Erythromycin | NR | 32.5  37.1  19.8 | | NR | 84 | NR | NR | Fig 3 | 0 | 0 | NR | 4.35 | 0 | 0 | 0 | 0 | NR | NR |
| Enrofloxacin | NR | 47.5  97.1  60.5 | | NR | NR | 0 | NR | 60  68.5 | NR | NR | NR | NR | NR | NR | NR | NR | 81.8 | NR |
| Tetracycline | NR | 62.5  62.6  60.4 | | NR | NR | 0  75 | NR | Fig 3 | 7.32 | 5.88 | NR | 2.90 | 28.57 | 0 | 100 | 100 | NR | NR |
| Nitrofurantoin | NR | 7.5  5.7  9.3 | | NR | NR | 0 | 54  11 | NR | NR | NR | NR | NR | NR | NR | NR | NR | NR | 31.2 |
| Trimethoprim-sulfamethoxazole | 12.5 | 22.5  22.9  11.6 | | NR | NR | 0  0 | 60 | Fig 3 | 7.32 | 0 | NR | 8.70 | 28.57 | 14.29 | 0 | 0 | NR | NR |
| Gentamicin | NR | 7.5  25.7  17.4 | | NR | NR | 0  0 | Fig 3 | Fig 3 | 100  7.32 | 6.7  5.88 | 19.4 | 4.35 | 0 | 14.29 | 0 | 0 | NR | 24.5 |
| Ceftazidime | NR | 5  2.9  3.5 | | 90 | NR | 0 | Fig 3 | Fig 3 | 93.3 | 0 | 9.7 | NR | NR | NR | NR | NR | NR | NR |
| Imipenem | NR | 45  48.6  47.7 | | NR | NR | 0 | Fig 3 | Fig 3 | 100 | 13.3 | 3.2 | NR | NR | NR | NR | NR | 0  3 | NR |
| Amikacin | NR | NR | | 0 | NR | 0 | Fig 3 | Fig 3 | 53.3 | 0 | 6.5 | NR | NR | NR | NR | NR | NR | 4.3 |
| Colistin | NR | NR | | NR | NR | 0 | NR | NR | NR | NR | NR | NR | NR | NR | NR | NR | NR | NR |
| Ertapenem | NR | NR | | 70 | NR | NR | NR | 0  0 | NR | NR | NR | NR | NR | NR | NR | NR | 0  3 | NR |
| Fosfomycin | NR | NR | | 0 | NR | NR | NR | NR | NR | NR | NR | NR | NR | NR | NR | NR | NR | NR |
| Tigecycline | NR | NR | | NR | NR | NR | 0 | NR | NR | NR | NR | NR | NR | NR | NR | NR | NR | NR |
| Cloxacillin | NR | NR | | NR | 78 | NR | NR | 4.4 | 0 | 17.65 | NR | 11.59 | 0.00 | 7.14 | 0 | 0 | NR | NR |
| Clindamycin | NR | NR | | NR | 69 | NR | NR | NR | NR | NR | NR | NR | NR | NR | NR | NR | NR | NR |
| Linezolid | NR | NR | | NR | 0 | NR | NR | NR | NR | NR | NR | NR | NR | NR | NR | NR | NR | NR |
| Vancomycin | NR | NR | | NR | 0 | NR | NR | NR | NR | NR | NR | NR | NR | NR | NR | NR | NR | NR |
| Ampicillin | NR | NR | | NR | NR | 0  75 | 100 | Fig3 | 34.15 | 23.53 | NR | 27.54 | 14.29 | 7.14 | 0 | 0 | NR | NR |
| Amoxicillin/Clavulanic acid | NR | NR | | NR | NR | 0 | NR | Fig 3 | NR | NR | NR | NR | NR | NR | NR | NR | NR | 62.8 |
| Neomycin | NR | NR | | NR | NR | 0 | NR | Fig 3 | NR | NR | NR | NR | NR | NR | NR | NR | NR | NR |
| Streptomycin | NR | NR | | NR | NR | 0  0 | NR | Fig 3 | NR | NR | NR | NR | NR | NR | NR | NR | NR | NR |
| Ceftriaxone | NR | NR | | NR | NR | 0 | Fig 3 | Fig 3 | NR | NR | NR | NR | NR | NR | NR | NR | NR | 44.5 |
| Norfloxacin | NR | NR | | NR | NR | NR | 85 | Fig 3 | NR | NR | NR | NR | NR | NR | NR | NR | NR | NR |
| Netilmicin | NR | NR | | NR | NR | NR | 6 | NR | NR | NR | NR | NR | NR | NR | NR | NR | NR | NR |
| Mecillinam | NR | NR | | NR | NR | NR | 9 | NR | NR | NR | NR | NR | NR | NR | NR | NR | NR | NR |
| Cefepime | NR | NR | | NR | NR | NR | 85.7  78.6 | NR | 69.7 | 0 | NR | NR | NR | NR | NR | NR | NR | NR |
| Cefazolin | NR | NR | | NR | NR | NR | NR | Fig 3 | NR | NR | NR | NR | NR | NR | NR | NR | NR | NR |
| Cefoxitin | NR | NR | | NR | NR | NR | NR | Fig 3 | NR | NR | NR | NR | NR | NR | NR | NR | NR | 9.8 |
| Kanamycin | NR | NR | | NR | NR | NR | NR | Fig 3 | NR | NR | NR | NR | NR | NR | NR | NR | NR | NR |
| Ofloxacin | NR | NR | | NR | NR | NR | NR | Fig 3 | NR | NR | NR | NR | NR | NR | NR | NR | NR | NR |
| Sulfadiazine | NR | NR | | NR | NR | NR | NR | 16.1 | 0 | 11.76 | NR | 8.70 | 0 | 0 | 0 | 0 | NR | NR |
| Azithromycin | NR | NR | | NR | NR | NR | NR | 5.8 | 0 | 0 | NR | 4.35 | 0 | 14.29 | 0 | 0 | NR | NR |
| Cephalexin | NR | NR | | NR | NR | NR | NR | 25.7  100 | NR | NR | NR | NR | NR | NR | NR | NR | 100 | NR |
| Ceftazidime/clavulanic acid | NR | NR | | NR | NR | NR | NR | 16.8 | NR | NR | NR | NR | NR | NR | NR | NR | NR | NR |
| Sulfamethoxazole | NR | NR | | NR | NR | NR | NR | Fig 3 | NR | NR | NR | NR | NR | NR | NR | NR | NR | NR |
| Trimethoprim | NR | NR | | NR | NR | NR | NR | Fig 3 | NR | NR | NR | NR | NR | NR | NR | NR | 63.6 | NR |
| Sulfonamide | NR | NR | | NR | NR | NR | NR | 42.8  0 | NR | NR | NR | NR | NR | NR | NR | NR | NR | NR |
| Carbapenem | NR | NR | | NR | NR | NR | NR | Fig 3 | NR | NR | NR | NR | NR | NR | NR | NR | 0  0.8  0 | NR |
| Oxytetracycline | NR | NR | | NR | NR | NR | NR | Fig 3 | NR | NR | NR | NR | NR | NR | NR | NR | 45.5 | NR |
| Piperacillin-tazobactam | NR | NR | | NR | NR | NR | Fig 3 | NR | 100 | 13.3 | 3.2 | NR | NR | NR | NR | NR | NR | 24.5 |
| Aztreonam | NR | NR | | NR | NR | NR | Fig 3 | NR | NR | 6.7 | 19.4 | NR | NR | NR | NR | NR | NR | 39.4 |
| Ticarcillin + Clavulanic acid | NR | NR | | NR | NR | NR | NR | NR | NR | NR | 32.3 | NR | NR | NR | NR | NR | NR | NR |
| Cefuroxime | NR | NR | | NR | NR | NR | NR | NR | NR | NR | NR | NR | NR | NR | NR | NR | NR | 48.9 |

ESBL: extended spectrum beta-lactamases, NR: Not reported, 0: Tested and reported as 0, Fig 3: Data graphically represented in Figure 03

Supplementary Data Table S2: Phenotypic antibiotic resistance (%) of Gram-positive bacteria to tested antibiotics

| **Antibiotic** | **MSSA** | **MRSA** | ***Streptococcus pneumoniae*** | ***Streptococcus agalactiae*** | ***Staphylococcus* spp.** | ***Streptococcus* spp.** | ***Micrococcus* spp.** | ***Bacillus* spp.** | ***Lactobacillus* spp.** | ***Streptomyces* spp.** | ***Listeria monocytogenes*** | ***Staphylococcus aureus*** | **Coagulase negative *Staphylococcus*** | **Coagulase positive *Staphylococcus*** |
| --- | --- | --- | --- | --- | --- | --- | --- | --- | --- | --- | --- | --- | --- | --- |
| Teicoplanin | 0  0 | 0  0 | NR | NR | NR | NR | NR | NR | NR | NR | NR | NR | NR | NR |
| Vancomycin | 0  0 | 0  0 | 0  0 | 0  0 | NR | NR | NR | NR | NR | NR | NR | NR | 0 | NR |
| Linezolid | 0  0  11.11 | Fig 3 | 0  0 | 0 | NR | NR | NR | NR | NR | NR | NR | 0 | 0 | NR |
| Doxycycline | 16.6  62.5 | 66.6  61.5 | NR | 56.7 | NR | NR | NR | NR | NR | NR | NR | NR | NR | 35.5 |
| Ampicillin | 76  88 | NR | NR | 0 | NR | 33.33 | 17.39 | 17.39 | 33.33 | 66.67 | 60 | NR | NR | 98.03 |
| Amoxicillin/Clavulanic acid | 12  16 | NR | NR | NR | NR | NR | NR | NR | NR | NR | NR | NR | NR | NR |
| Ciprofloxacin | 13.3  60  51.3  26.3 | Fig 3 | NR | 0 | 5.71  7.1 | 13.33 | 13.04 | 10.14 | 16.67 | 0 | NR | 55.6 | 58 | 0 |
| Clindamycin | 85  100  54.16  12.3 | Fig 3 | 69.2 | 0  33.3 | NR | NR | NR | NR | NR | NR | NR | 44.4 | 69 | NR |
| Erythromycin | 65.27  57.9 | Fig 3 | 80.8  74.8 | 15  37.7 | 2.86 | 13.33 | 0 | 7.25 | 16.67 | 0 | NR | 66.7 | 84 | NR |
| Gentamicin | 32.3  44.3  23.61  5.3 | Fig 3 | NR | 100 | 2.86 | 0 | 0 | 4.35 | 0 | 0 | NR | NR | NR | NR |
| Trimethoprim-sulfamethoxazole | 3.6  6.6  19.44 | Fig 3 | 65.3 | NR | 8.57 | 0 | 8.7 | 5.8 | 0 | 0 | NR | NR | NR | 35.3 |
| Chloramphenicol | 8.6  7.2  9.72 | Fig 3 | NR | 0 | 50 | NR | NR | NR | NR | NR | NR | NR | NR | 45.1 |
| Fusidic acid | 0  0  14 | Fig 3 | NR | NR | NR | NR | NR | NR | NR | NR | NR | NR | NR | NR |
| Cefuroxime | 32  34 | NR | NR | NR | NR | NR | NR | NR | NR | NR | NR | NR | NR | NR |
| Cefoxitin | 43.05 | 100 | NR | NR | NR | NR | NR | NR | NR | NR | NR | NR | NR | NR |
| Amikacin | 18.04 | 32.25 | NR | NR | NR | NR | NR | NR | NR | NR | NR | NR | NR | 68.6 |
| Tetracycline | 30.54 | Fig 3 | 80.8  74.8 | 58.3 | 0 | 0 | 8.7 | 5.8 | 25 | 0 | 14 | NR | NR | NR |
| Penicillin | 87.5 | 100 | 26.9  5.6  92.5 | 0  0 | NR | NR | NR | NR | NR | NR | 40 | NR | NR | NR |
| Enrofloxacin | NR | Fig 3 | NR | NR | 0 | NR | NR | NR | NR | NR | NR | NR | NR | NR |
| Kanamycin | NR | Fig 3 | NR | NR | NR | NR | NR | NR | NR | NR | NR | NR | NR | 43.1 |
| Neomycin | NR | Fig 3 | NR | NR | NR | NR | NR | NR | NR | NR | NR | NR | NR | 54.9 |
| Nitrofurantoin | NR | Fig 3 | NR | NR | NR | NR | NR | NR | NR | NR | NR | NR | NR | NR |
| Rifampicin | NR | Fig 3 | 0 |  | NR | NR | NR | NR | NR | NR | NR | NR | NR | NR |
| Cefotaxime | NR | NR | 3.8  17.7  72 | 0  0 | NR | NR | NR | NR | NR | NR | NR | NR | NR | NR |
| Meropenem | NR | NR | 73 | NR | NR | 40 | 30.43 | 20.29 | 33.33 | 0 | NR | NR | NR | NR |
| Amoxycillin | NR | NR | 7.8 | NR | 20 | NR | NR | NR | NR | NR | NR | NR | NR | 64.7 |
| Cefepime | NR | NR | 57.7 | NR | NR | NR | NR | NR | NR | NR | NR | NR | NR | NR |
| Levofloxacin | NR | NR | 0  0 | 0 | NR | NR | NR | NR | NR | NR | NR | NR | NR | NR |
| Oxacillin | NR | NR | NR | 0 | NR | NR | NR | NR | NR | NR | NR | NR | NR | NR |
| Ceftibuten | NR | NR | NR | 0 | NR | NR | NR | NR | NR | NR | NR | NR | NR | NR |
| Minocycline | NR | NR | NR | 56.7 | NR | NR | NR | NR | NR | NR | NR | NR | NR | NR |
| Lincomycin | NR | NR | NR | 0 | NR | NR | NR | NR | NR | NR | NR | NR | NR | NR |
| Gatifloxacin | NR | NR | NR | 0 | NR | NR | NR | NR | NR | NR | NR | NR | NR | NR |
| Moxifloxacin | NR | NR | NR | 0 | NR | NR | NR | NR | NR | NR | NR | NR | NR | NR |
| Trimethoprim | NR | NR | NR | NR | 57.1 | NR | NR | NR | NR | NR | NR | NR | NR | 52.9 |
| Oxytetracycline | NR | NR | NR | NR | 57.1 | NR | NR | NR | NR | NR | NR | NR | NR | 56.8 |
| Cephalexin | NR | NR | NR | NR | 92.9 | NR | NR | NR | NR | NR | NR | NR | NR | NR |
| Cloxacillin | NR | NR | NR | NR | 14.29  21.8 | 0 | 0 | 14.49 | 0 | 33.33 | NR | 88.9 | 78 | 64.7 |
| Sulfadiazine | NR | NR | NR | NR | 2.86 | 0 | 30.43 | 7.25 | 25 | 0 | NR | NR | NR | NR |
| Azithromycin | NR | NR | NR | NR | 0 | 0 | 0 | 7.25 | 0 | 0 | NR | NR | NR | NR |
| Streptomycin | NR | NR | NR | NR | NR | NR | NR | NR | NR | NR | 34 | NR | NR | NR |

MSSA: Methicillin-Sensitive *Staphylococcus aureus*, MRSA: Methicillin-resistant *Staphylococcus aureus*, NR: Not reported, 0: Tested and reported as 0, Fig 3: Data graphically presented in Figure 03
